# Isolation of Multidrug-Resistant *Escherichia coli* O157 from Goats in the Somali Region of Ethiopia: A Cross-Sectional, Abattoir-Based Study

**DOI:** 10.1371/journal.pone.0142905

**Published:** 2015-11-11

**Authors:** Fitsum Dulo, Aklilu Feleke, Barbara Szonyi, Reinhard Fries, Maximilian P. O. Baumann, Delia Grace

**Affiliations:** 1 Faculty of Veterinary Medicine, Addis Ababa University, Debre Zeit, Ethiopia; 2 International Livestock Research Institute, Addis Ababa, Ethiopia; 3 Institute of Meat Hygiene and Technology, Freie Universität Berlin, Berlin, Germany; 4 FAO Reference Centre for Veterinary Public Health (VPH), Freie Universität Berlin, Berlin, Germany; 5 International Livestock Research Institute, Nairobi, Kenya; Universidade de Aveiro, PORTUGAL

## Abstract

Toxigenic *Escherichia coli (E*. *coli)* are an important cause of gastroenteritis in developing countries. In Ethiopia, gastroenteritis due to food-borne disease is a leading cause of death. Yet, there is no surveillance for *E*. *coli* O157 and little is known about the carriage of this pathogen in Ethiopia’s livestock. This study aimed to assess the prevalence and levels of antimicrobial resistance of *E*. *coli* O157 in goat meat, feces, and environmental samples collected at a large abattoir in the Somali region of Ethiopia. The samples were enriched in modified tryptone broth containing novobiocin, and plated onto sorbitol MacConkey agar. Isolates were confirmed using indole test and latex agglutination. Antimicrobial susceptibility testing was conducted using the disk diffusion method. A total of 235 samples, including 93 goat carcass swabs, 93 cecal contents, 14 water, 20 hand, and 15 knife swabs were collected. Overall, six (2.5%) samples were contaminated with *E*. *coli* O157 of which two (2.1%) were isolated from cecal contents, three (3.2%) from carcass swabs, and one (7.1%) from water. All isolates were resistant to at least two of the 18 antimicrobials tested. Two isolates (33.3%) were resistant to more than five antimicrobials. Abattoir facilities and slaughter techniques were conducive to carcass contamination. This study highlights how poor hygiene and slaughter practice can result in contaminated meat, which is especially risky in Ethiopia because of the common practice of eating raw meat. We detect multi-resistance to drugs not used in goats, suggesting that drugs used to treat human infections may be the originators of antimicrobial resistance in livestock in this ecosystem. The isolation of multidrug-resistant *E*. *coli* O157 from goats from a remote pastoralist system highlights the need for global action on regulating and monitoring antimicrobial use in both human and animal populations.

## Introduction

*Escherichia coli (E*. *coli)* are a group of bacteria that are part of the intestinal micro-flora of healthy animals and humans. However, certain serogroups, including *E*. *coli* O157, can carry genes that allow them to produce toxins known as verotoxins or Shiga-like toxins. Verotoxigenic *E*. *coli* (VTEC) are not pathogenic to ruminants, but they cause serious diseases in humans worldwide, including diarrhea, hemorrhagic colitis, hemolytic-uremic syndrome, and sometimes death [1]. Consumption of contaminated raw and undercooked meat is the most common means of transmission to humans [2].

Domestic ruminants, including goats, are natural reservoirs for *E*. *coli* O157; therefore they play a significant role in the epidemiology of human infections [3]. The pathogen is carried in the intestinal tract and excreted in the feces. During slaughter, the pathogen may be present on the skin or in the feces of the animal, and may get transferred to the carcass during evisceration or skin removal. Therefore, poor slaughter techniques, particularly poor hygienic practices during slaughter greatly increase the risk of meat contamination with *E*. *coli* O157. The risk of meat contamination also depends on the *E*. *coli* O157 carriage status of the slaughter animals [4]. Therefore, assessment of slaughter hygiene and the carriage status of the pre-slaughter animal population are essential in determining the risk of exposure of meat consumers to *E*. *coli* O157.

Additionally, antimicrobial resistance among enteric bacteria is an increasing global public health concern. The widespread administration of antimicrobials promotes the selection of antimicrobial resistant strains, which complicates the treatment of bacterial infections. Furthermore, antimicrobial resistance also has negative effects on animal health and the environment as per the One Health concept which highlights the interconnection of human, animal, and environmental health. Livestock in particular, are often considered as sources of antimicrobial resistance in industrialized countries, where antimicrobials are commonly used to improve productivity [5].

Gastroenteritis due to food-borne disease is one of the most common illnesses in Ethiopia, and it is a leading cause of death among people of all ages in the country [6]. The lack of surveillance of food-borne pathogens, poor hygienic conditions and sub-standard slaughter practices in the abattoirs, and the widespread cultural practice of raw meat consumption, are all major factors contributing to the high risk of exposure of Ethiopians to food-borne pathogens such as VTEC. In spite of the high risk of exposure to VTEC in Ethiopia, there is no surveillance for this pathogen and very little is known about the carriage rate of *E*. *coli* O157 in different livestock populations. Also, more information is needed on antimicrobial resistance patterns in developing countries like Ethiopia, where both veterinary and medical drugs are often misused, creating ideal conditions for the development of resistant strains. To reduce this knowledge gap, this study was carried out to 1) assess the pre-slaughter (*i*.*e*. carriage status) of *E*. *coli* O157 in goats originating in the Somali region of Ethiopia; 2) to assess the hygienic practices and the level of carcass contamination with *E*. *coli* O157 during the slaughter process of goats and 3) to determine the antimicrobial susceptibility pattern of the isolates.

## Materials and Methods

### Description of study area

The target population for this study was goats (*Capra aegagrus hircus*) raised by pastoralists in the Somali region of Ethiopia. The arid Somali zone covers over 250,000 square kilometers in the eastern part of Ethiopia (S1 Fig) (http://reliefweb.int/map/ethiopia/administrative-regions-ethiopia; Accessed September 15^th^ 2015). It is greater than the size of France and is home to over 5 million people, 38% of whom are pastoralists [7]. The goats kept by pastoralists are indigenous breeds (i.e. long-eared Somali goats). The average herd size is 8–12 goats. Goat production in the study area relies on browsing on natural pasture. Goats share common pastures with sheep, cattle and camels. Shortage of feed due to frequent drought is a major challenge and pastoralists use seasonal migration to cope with the dry conditions. Pastoralists in the study area house their livestock at night using fences collected from the bush [8]. The study abattoir is located in a large industrial city within the Somali region, which serves as a main destination for slaughter animals particularly from the Shinille Zone. The study abattoir is among the three largest abattoirs in the Somali region, and has a maximum capacity to slaughter approximately 350 goats per week; they also slaughtered sheep, cattle and camel. The demand for meat widely fluctuates throughout the year, with sharp increases in demand during religious holidays. The number of employees in the abattoir also fluctuates to accommodate the demand, with the highest number of workers hired during peak seasons. At the time of study, there were 20 employees in the abattoir.

### Study design

A cross-sectional study was conducted in 2014 in one of the largest abattoirs in the Somali zone of Ethiopia, with the goal to determine the prevalence and antimicrobial resistance patterns of *E*. *coli* O157 in goat meat, feces, and environmental samples. Sampling was carried out over a period of 2 months on seven different occasions until the desired sample size was reached. At each visit, animals were selected using systematic random sampling method. This sampling approach was designed to minimize the chance of sampling multiple animals from the same herd. All goats originated from the Somali region of Ethiopia.

Sample size was determined using the formula by Thrusfield [9], based on expected prevalence of *E*. *coli* O157 in goat meat and feces, which was estimated at 5% following Mersha *et al*. [10]. The confidence level was 95% and the precision was 5%. Thus, the required sample size was 73; however 93 samples were taken deliberately in order to maximize the precision of the study.

### Sample collection

#### Carcass sampling

A total of 93 carcass swabs were collected. For each animal, four different sites of the carcass (thorax, brisket, flank and crutch) were swabbed following the guidelines of the International Organization for Standardization (ISO)[11]. Each sampling area covered 100 cm^2^ by placing a sterile template (10 cm x 10 cm) on the carcass. For each sampling area, a sterile cotton-tipped swab (2 X 3 cm), fitted with shaft, was first moistened in 10 ml of buffered peptone water (Oxoid Ltd., Hampshire, UK) and then rubbed first horizontally and then vertically several times across the carcass surface. On completion of the rubbing process, the shaft was broken by pressing it against the inner wall of the test tube and disposed leaving the cotton swab in the test tube. Different swabs were used on the four different sites, but they were pooled. The four swabs were put into a screw-capped test tube containing 10 ml of sterile buffer peptone water, and were transported to the laboratory in a cool box on ice within twenty four hours of sampling.

#### Cecal sampling

A total of 93 cecal contents were collected. The cecal samples were collected immediately after evisceration from cecal contents of slaughtered goats; an aseptic incision was made with surgical blade in the cecum to obtain a representative sample of 10 g of the cecal content. The fecal material was aseptically compressed and the resultant liquid was decanted in sterile universal bottle. The samples were labeled and transported in an icebox on ice to the laboratory, where they were held in a cold storage (2–3°C) overnight and processed the following day.

#### Environmental sampling and assessment of slaughter hygiene

A total of 49 environmental samples were collected, consisting of 14 water samples, 20 hand swabs and 15 butcher knife swabs. Environmental samples were collected by swabbing the hands of abattoir workers (both hands, both sides) and swabbing the slaughter knives (blade and handle, both sides). In addition, water samples (10 ml) were collected before and during operation from the water buckets that were used for cleaning equipment and washing hands. Slaughtering hygiene and the sanitary status of the abattoir were determined by the use of a structured checklist and through direct observations of the premises and the practices of abattoir workers. The observational survey was conducted over the course of seven visits. At each visit, two workers were observed. Overall, 14 workers were observed. Each worker was observed for 30 minutes during operation, resulting in a total of 7 hours of observation time. Each worker’s hands were only swabbed once, during operation. At least two knife swabs and two water samples were taken during each of the seven visits. Carcass swabs and cecal samples were also taken at each visit.

Written permission was obtained from the management of the study abattoir to conduct the study. A standard consent form was read out to all potential study participants. Verbal consent was obtained because some of the participants were illiterate. If a potential participant did not agree to take part in the study, they were not included in the study. If a participant agreed, then a box was ticked on the coded questionnaire noting that the participant has given consent. The management served as witnesses. The study overall was approved by the Institutional Research Ethics Committee of the International Livestock Research Institute (Ref. No. IREC-2013-03), the Academic Commission of the College of Veterinary Medicine and Agriculture, Addis Ababa University, and the National research Ethics Review Committee (NRERC) of the Ministry of Science and Technology of the Federal Democratic Republic of Ethiopia (Ref No 3-30/773-06).

Based on the results of this survey, we compiled a training package on hygienic practices for abattoir workers. The training has been delivered to workers of selected abattoirs, including to workers of the study abattoir, in Ethiopia. The results of the study were also conveyed during the training session.

### Culture and isolation of *E*. *coli* O157

#### Fecal samples

Approximately 1ml/1g of fecal pellet (homogenized when possible) was suspended into 9 ml of modified tryptone soya broth supplemented with novobiocin (10 mg/l) (Oxoid Ltd, Hampshire, UK). Samples were vortexed and incubated overnight at 37°C. After selective enrichment, 50μl of product was streaked onto sorbitol MacConkey agar (Oxoid Ltd., Hampshire, UK) and the plates were incubated at 37°C for 24 hours. Up to six colorless colonies (non- sorbitol fermenters) were picked and separately sub-cultured on MacConkey agar for 24 hours at 37°C for purification. After overnight incubation, the purified and intensely red colonies with a pale periphery were tested for indole production and indole forming isolates were seeded onto nutrient agar for serological confirmation by latex agglutination. The indole test was carried out as follows. One colony was inoculated into 4 ml of tryptone soya broth, using a straight inoculation wire. Incubation was done for overnight at 37°C. After this, one drop of indole reagent was added to the tryptone soya broth culture to test for indole production (red ring-positive). The microbiological analysis was carried out at the Ethiopian Nutrition and Health Research Institute (EHNRI).

#### Carcass bacterial swabs and environmental samples

The carcass bacterial swabs and the environmental samples were incubated overnight at 37°C after being suspended into modified tryptone soya broth supplemented with novobiocin (1:9), and subjected to similar tests for bacteriological analysis as fecal samples.

Confirmatory test by latex agglutination for E. coli O157 serogroup

Non-sorbitol fermenting (NSF) isolates were inoculated onto nutrient agar for testing. Then, the serogroup of NSF and indole positive colonies was identified using the DrySpot *E*. *coli* O157 latex agglutination test (Oxoid Ltd., Hampshire, UK). One drop of saline was dispensed to the small ring (at the bottom of each oval) in both the test and control reaction areas ensuring that the liquid did not mix with the dried latex reagents. Using a sterile single use plastic loop, a portion of the colony to be tested was picked and carefully emulsified in the saline drop until the suspension was smooth. Then, using paddle the suspension was mixed into the dry latex spots until completely suspended and spread to cover the reaction area. The test card was picked up and rocked for up to 60 seconds, and agglutination was detected under normal lighting conditions. A result was positive if agglutination of the latex particles occurred within 1 minute. This indicated the presence of *E*. *coli* serogroup O157. A negative result was obtained if no agglutination occurred and a smooth blue suspension remained after 60 seconds in the test area.

### Antimicrobial susceptibility testing

Antimicrobial resistance tests were performed by standard disc diffusion technique [12]. The selection criteria of antimicrobials depended on the frequency of use of antimicrobials in the ruminants, potential public health importance and recommendations from the guideline of antimicrobial susceptibility testing from the Clinical and Laboratory Standards Institute (CLSI) [12].

Resistance testing discs included 18 antimicrobial agents (Oxoid Ltd., Hampshire, UK) as listed in Table 1. The isolates were considered resistant if the diameter of the inhibition zone was less than or equal to the resistance breakpoint provided by CLSI guidelines.

**Table 1:** Antimicrobials used and interpretation of resistance.

Antimicrobial disc	Code	Concentration (μg)	Diameter of zone of inhibition (mm)		
			Resistant (≤)	Intermediate	Susceptible (≥)
**Ampicillin**	AMP	10	13	14–16	17
**Amoxycillin-Clavulanic acid**	AMC	20/10	13	14–17	18
**Cefotaxime**	CTX	30	22	23–35	26
**Ceftriaxone**	CRO	30	19	20–22	23
**Cefoxitin**	FOX	30	14	15–17	18
**Cefuroxime Sodium**	CXM	30	14	15–17	18
**Chloramphenicol**	C	30	12	13–17	18
**Ciprofloxacin**	CIP	5	15	16–20	21
**Erythromycin**	E	15	13	14–22	23
**Gentamicin**	CN	10	12	13–14	15
**Kanamycin**	K	30	13	14–17	18
**Nalidixic acid**	NA	30	13	14–18	19
**Nitrofurantoin**	F	50	14	15–16	17
**Norfloxacin**	NOR	10	12	13–16	17
**Streptomycin**	S	10	11	12–14	15
**Sulfamethoxazole**	SXT	25	10	11–15	16
**Trimethoprim-Sulfonamides**	S3	300	12	13–16	17
**Tetracycline**	TE	30	11	12–14	15

Each isolated bacterial colony from pure fresh culture was transferred into a test tube of 5 ml tryptone soya broth and incubated at 37°C for 6 hours. The turbidity of the culture broth was adjusted using sterile saline solution, or more isolated colonies were added to obtain turbidity that is usually comparable with that of 0.5 McFarland standards (approximately 3x10^8^ CFU per ml). Mueller-Hinton agar (Becton, Dickinson and Company, New Jersey, USA) plates were prepared according to the manufacturer. A sterile cotton swab was immersed into the suspension and rotated against the side of the tube to remove the excess fluid and then swabbed in three directions uniformly on the surface of Mueller-Hinton agar plates. After the plates dried, antimicrobial disks were placed on the inoculated plates using sterile forceps. The antimicrobial disks were gently pressed onto the agar to ensure firm contact with the agar surface, and incubated at 37°C for 24 hours. Following this, the diameter of inhibition zone formed around each disk was measured using a black surface, reflected light and transparent ruler by laying it over the plates.

### Data analysis

Data were transferred to a Microsoft Excel spread sheet (Microsoft Corp., Redmond, WA, USA). The prevalence of *E*. *coli* O157 in cecal contents, carcass swab and environmental samples was determined by dividing the number of positive samples by the total number of samples examined. The significance of association between *E*. *coli* O157 carriage and type of sample (cecal content *vs*. carcass) was assessed using the Fisher’s exact test in SPSS 20 statistical software (SPSS Inc., Chicago, IL, USA); differences were considered significant at *P* < 0.05.

## Results

### Isolation and prevalence of *E*. *coli* O157

Out of the total of 235 different samples examined, six (2.6%) were found to be contaminated with *E*. *coli* O157. *Escherichia coli* O157 was isolated from two (2.2%) cecal contents, three (3.2%) carcass swabs, and one (7.1%) water sample. One animal was positive in both cecal contents and carcass swabs. The water samples could not be associated with specific animals, because the same buckets were used to clean hands and equipment in the process of slaughtering multiple animals. There was no statistically significant difference in the proportion of positive isolates between cecal contents and carcass swabs.

### Susceptibility of isolates to antimicrobial agents

Antimicrobial susceptibility testing results showed that of the 6 isolates, all were resistant to erythromycin, 5(83.3%) were resistant to ampicillin, 3(50%) were resistant to nitrofurantoin, 2(33.3%) were resistant to cefoxitin, streptomycin, sulfamethoxazole-trimethoprim, sulfonamides and tetracycline, and 1(16.7%) showed resistance to amoxycillin-clavulanic acid. None of the isolates were resistant to cefotaxime, ceftriaxone, cefuroxime sodium, chloramphenicol, ciprofloxacin, gentamicin, nalidixic acid and norfloxacin. All isolates were resistant to at least two antimicrobials. Two isolates (33.3%) were resistant to more than five antimicrobials tested (Table 2).

**Table 2:** Antimicrobial resistance patterns of *E*. *coli* O157 isolates.

Isolate type	^a^Resistance patterns
water	AMP,E
carcass	E,F
carcass	AMP,E,F
carcass	AMP,FOX,E
cecum	AMP,E,S,SXT,S3,TE
cecum	AMP,AMC,E,F,FOX,S,SXT,S3,TE

^a^Key for Table 2: AMP: ampicillin, AMC: amoxycillin-clavulanic acid, FOX: cefoxitin, E: erythromycin, F: nitrofurantoin, S: streptomycin, SXT: sulfamethoxazole-trimethoprim, S3: sulfonamides, TE: tetracycline

### Facilities and practices at the abattoir

The observational survey revealed that deficiencies in the abattoir’s facilities did not allow for maintenance of hygiene (Table 3). For example, tap water, hot water, soap, retention room (cooling facilities), pest control, changing rooms, latrine and bathroom facilities were not present in the abattoir. The entire slaughter process was done in the same area without separation of dirty and clean zones. Water was only available in buckets, and the same buckets of water were used for cleaning knives, washing hands, washing carcasses, and washing the floor. The workers placed their equipment on dirty surfaces including the floor during their work. Other risky practices, such as “fisting” were also observed (14/14; 100%). During “fisting”, the butcher punches his fist forcefully between the skin and the carcass surface to detach the skin. A meat inspector veterinarian was always present.

**Table 3:** Checklist and results of observational survey on abattoir hygiene.

Workers’ hygiene	Use of protective clothing	Gown -14/14 (100%)
	Protective clothing is clean	2/14 (14%)
	Cuts/wounds covered with waterproof dressing	2/4 (50%)
	Means of washing and disinfection of personal slaughter equipment	Plain water in buckets14/14 (100%)
	Slaughter knife free from damages and dirt	0/14 (0%)
	Slaughter knife rested on clean surface during carcass processing	0/14 (0%)
	Regular hand washing during work (before, during and after processing each carcass)	0/14 (0%)
	Received job-related training on hygiene	3/14 (21.4%)
**Toilet facilities**	Latrine/toilet on premises	Absent
	Water in latrine	Not applicable
	Soap in latrine	Not applicable
	Tissue paper in latrine	Not applicable
	Paper towel in latrine	Not applicable
**Slaughter hall hygiene**	Meat inspector veterinarian	Present
	Rodent and insect control	Absent
	Ventilation	Inadequate
	Running water in slaughter hall	Absent
	Soap in slaughter hall	Absent
	Disinfectant in slaughter hall	Absent
	Electricity/lighting	Inadequate
	Separation of clean and dirty areas	Absent

## Discussion

To our knowledge, this was the first study to investigate the presence, prevalence, and antimicrobial resistance patterns of *E*. *coli* O157 in goats slaughtered in a major abattoir in the remote Somali region of Ethiopia. We isolated *E*. *coli* O157 from 3.2% of carcass swab samples, 2.2% of cecal contents and 7.1% of water samples. The hygienic conditions and slaughter practices in the abattoir were found to be conducive to cross-contamination of goat carcasses with pathogens including *E*. *coli* O157, posing a risk to meat consumers.

The prevalence of *E*. *coli* O157 in carcass swabs in the current study is consistent with findings of previous studies conducted in other regions of Ethiopia. Mersha et al. [10] in Modjo, and Hiko et al. [13] in Debre Zeit, reported a prevalence of *E*. *coli* O157 in 3% and 5% of goat meat samples, respectively. According to current international guidelines, any detection of *E*. *coli* O157 in meat is considered unacceptable (http://www.fsis.usda.gov/OPPDE/rdad/FRPubs/99-1123.htm; accessed September 4^th^, 2015). However, there are no regulations in Ethiopia to protect meat consumers from food-borne pathogens such as *E*. *coli* O157. This is especially problematic because of the widespread practice of raw meat consumption throughout the country. Therefore, avoiding carcass contamination during the slaughter process should be of utmost importance in order to protect the public from food-borne illness due to consumption of contaminated meat.

In the present study, the Somali region of Ethiopia was chosen because of its large goat population. According to official statistics there are at least 3 million goats in this area [14]. Much of the zone is remote and isolated but it is a major supplier of goat meat to both domestic markets and for export to Djibouti, Somalia and the Middle East [8]. Furthermore, the consumption of goat meat is much more frequent in the Somali region compared to other areas of Ethiopia, with fresh goat meat reportedly purchased by the predominantly Muslim population from retail shops 1–5 times a week. In the Somali region, goats are raised by pastoralists who have frequent, close contact with their animals. Thus, the risk of transmission of zoonotic diseases in this system is high. Furthermore, the deeply-rooted cultural practice of raw meat consumption throughout Ethiopia increases the risk of food-borne illness. This, combined with our results suggests that food-borne illness due to *E*. *coli* O157 could be a significant public health concern in the Somali region of Ethiopia and possibly in other parts of the country as well. Clinical data are needed to estimate the real impact of *E*. *coli* O157 on human health in Ethiopia.

In this study, *E*. *coli* O157 was isolated from both cecal contents and carcass swabs.

The presence of *E*. *coli* O157 on goat carcasses indicates transfer of fecal material onto the sterile carcass during the slaughter process. Indeed, risky practices during the slaughtering process were observed and documented in the present study. For instance, the practice of fisting, whereby the workers touch the soiled outside of the animal’s skin while removing the skin, can facilitate transfer of pathogens onto the sterile carcass surface. Fisting should be done with utmost care to avoid carrying dirt, debris and pathogens from the skin onto the sterile carcass surface, but butchers were observed touching both the inside of the carcass and the soiled outside of the animal’s skin while removing the skin. We also isolated *E*. *coli* O157 from abattoir water. In the abattoir, water was stored in plastic buckets and the same bucket was used to wash the floor, carcasses, hands and equipment. Therefore, wash water in this abattoir could be a major source of contamination, and could contribute to carcass-to-carcass spread of pathogenic bacteria across the slaughter line. The survey also revealed that workers at the abattoir did not have adequate training in safe meat handling and proper slaughtering hygiene. Also, they were not equipped and supplied with the necessary materials that would enable them to maintain general hygiene. For instance, some of the slaughter staff indicated that inadequate supply of clean water posed a challenge towards maintaining hygiene. These are common problems in public abattoirs throughout Ethiopia. Often, the resources allocated to public abattoirs are not sufficient to properly train staff and to maintain equipment and facilities in hygienic conditions. To address some of these issues, our team has been collaborating with the Ministry of Agriculture on providing training to abattoir workers on proper practices and hygiene during the slaughter process.

All *E*. *coli* O157 isolates in the present study exhibited resistance to at least two or more of the 18 antimicrobial agents tested. Resistance to erythromycin and ampicillin were the most common resistance profiles identified among our study isolates. High resistance of *E*. *coli* O157 to erythromycin is common and has also been reported from the Middle East [15] [16]. Furthermore, half of the *E*. *coli* O157 isolates were resistant to nitrofurantoin. Interestingly, there was only moderate resistance to tetracycline in this study, although it is one of the most commonly available drugs for use among livestock in Ethiopia. It is readily available in different dosage forms and in combination with other antimicrobials and vitamins. To our knowledge, this is the only antimicrobial drug which is most likely to be readily available in the study area for veterinary use. Interestingly, we detected resistance to cefoxitin, which is thought to be uncommon in *E*. *coli*; however, it has been reported from India [17] and Canada [18]. No resistance was observed to the newer generation of antimicrobials such as ciprofloxacin and norfloxacin, which are important in the treatment of human cases of gastroenteritis.

In the Somali zone of Ethiopia, pastoralists rear goats in an extensive system without the use of specialized inputs. Here, goats only feed on natural forages and shrubs, veterinary input is minimal, and the availability of veterinary drugs is limited [8]. However, the human population in this region has access to antimicrobials, albeit often through informal channels with neither diagnosis nor proper recommendations for use. Such misuse is conducive to the rise of antimicrobial resistance; thus the origin of resistance may have been drugs used to treat human infections. Alternatively, natural antimicrobial resistance may explain some of the observed resistance. Further investigations are needed to determine which factors are responsible for the emergence of multidrug-resistant strains in this vast and extensive production system.

The present study had some limitations in laboratory facility and therefore, other serogroups and virulence factors for *E*.*coli* O157 were not investigated. Also, it has been shown that the use of immune-magnetic separation (IMS) with enrichment in broth culture enhances the isolation of *E*. *coli* O157 from samples with a low concentration of the bacteria [19]. In this study, enrichment without IMS was employed for the isolation of *E*. *coli* O157. Nonetheless, the present study revealed that multidrug-resistant *E*. *coli* O157 were present in the goat population of the remote Somali region of Ethiopia, which emphasizes the need for more stringent monitoring of antimicrobial use in both human and animal populations.

## Supporting Information

S1 Fig Map of Ethiopia with administrative regions. (PDF)

## References

[1] Farrokh C, Jordan K, Auvray F, Glass K, Oppegaard H, Raynaud S, et al Review of Shiga-toxin-producing *Escherichia coli* (STEC) and their significance in dairy production. Int J Food Microbiol. 2012;162(2):190–212. doi: 10.1016/j.ijfoodmicro.2012.08.008 22939912 10.1016/j.ijfoodmicro.2012.08.008

[2] Chapman PA, Cerdan Malo AT, Ellin M, Ashton R, Harkin. *Escherichia coli* O157 in cattle and sheep at slaughter, on beef and lamb carcasses and in raw beef and lamb products in South Yorkshire, UK. Int J Food Microbiol. 2001;64(1–2):139–50. . 11252496 10.1016/s0168-1605(00)00453-0

[3] Griffin PM, Tauxe RV. The epidemiology of infections caused by *Escherichia coli* O157:H7, other enterohemorrhagic *E*. *coli*, and the associated hemolytic uremic syndrome. Epidemiol Rev. 1991;13:60–98. . 1765120 10.1093/oxfordjournals.epirev.a036079

[4] McEvoy JM, Doherty AM, Sheridan JJ, Thomson-Carter FM, Garvey P, McGuire L, et al The prevalence and spread of *Escherichia coli* O157:H7 at a commercial beef abattoir. J Appl Microbiol. 2003;95(2):256–66. . 12859756 10.1046/j.1365-2672.2003.01981.x

[5] Van Boeckel TP, Brower C, Gilbert M, Grenfell BT, Levin SA, Robinson TP, et al Global trends in antimicrobial use in food animals. Proc Natl Acad Sci USA. 2015 . 25792457 10.1073/pnas.1503141112 PMC4426470

[6] Institute for Health Metrics and Evaluations (IHME). Global Burden of Disease Study 2010 Seattle, WA: University of Washington; 2013.

[7] Central Statistical Authority. Census 2007 Tables: Somali Region. Addis Ababa, Ethiopia: Ministry of Finance and Economic Development, 2007.

[8] Hassen A, Ismail A, Haile A, Legese G. Analysis of sheep and goat value chains in Shinille District, Somali Region, Ethiopia Addis Ababa: ICARDA, 2013.

[9] Thrusfield M. Veterinary Epidemiology. 3rd ed. Ames, Iowa: Blackwell Publishing Professional; 2005.

[10] Mersha G, Asrat D, Zewde BM, Kyule M. Occurrence of *Escherichia coli* O157:H7 in faeces, skin and carcasses from sheep and goats in Ethiopia. Lett Appl Microbiol. 2009;50(1):71–6. . 19895421 10.1111/j.1472-765X.2009.02757.x

[11] International Organization for Standardization (ISO). Microbiology of food and animal feeding stuffs -carcass sampling for microbiological analysis Geneva, Switzerland: ISO; 2013.

[12] Clinical and Laboratory Standards Institute (CLSI). Performance standards for antimicrobial susceptibility testing; Twenty-second informational supplement Wayne, PA: CLSI; 2012.

[13] Hiko A, Asrat D, Zewde G. Occurrence of *Escherichia coli* O157:H7 in retail raw meat products in Ethiopia. J Infect Dev Ctries. 2008;2(5):389–93. . 19745509 10.3855/jidc.203

[14] Central Statistical Authority. Livestock aerial survey in the Somali region Addis Ababa, Ethiopia: Ministry of Finance and Economic Development, 2003.

[15] Harakeh S, Yassine H, Gharios M, Barbour E, Hajjar S, El-Fadel M, et al Isolation, molecular characterization and antimicrobial resistance patterns of *Salmonella* and *Escherichia coli* isolates from meat-based fast food in Lebanon. Sci Total Environ. 2005;341(1–3):33–44. . 15833239 10.1016/j.scitotenv.2004.09.025

[16] Osaili TM, Alaboudi AR, Rahahlah M. Prevalence and antimicrobial susceptibility of *Escherichia coli* O157:H7 on beef cattle slaughtered in Amman abattoir. Meat Sci. 2013;93(3):463–8. doi: 10.1016/j.meatsci.2012.11.023 23273451 10.1016/j.meatsci.2012.11.023

[17] Shahid M, Malik A, Akram M, Agrawal LM, Khan AU, Agrawal M. Prevalent phenotypes and antibiotic resistance in *Escherichia coli* and *Klebsiella pneumoniae* at an Indian tertiary care hospital: plasmid-mediated cefoxitin resistance. Int J Infect Dis. 2008;12(3):256–64. . 17981482 10.1016/j.ijid.2007.08.008

[18] Mulvey MR, Bryce E, Boyd DA, Ofner-Agostini M, Land AM, Simor AE, et al Molecular characterization of cefoxitin-resistant *Escherichia coli* from Canadian hospitals. Antimicrob Agents Chemother. 2005;49(1):358–65. . 15616316 10.1128/AAC.49.1.358-365.2005 PMC538860

[19] Chapman PA, Wright DJ, Siddons CA. A comparison of immunomagnetic separation and direct culture for the isolation of verocytotoxin-producing *Escherichia coli* O157 from bovine faeces. J Med Microbiol. 1994;40(6):424–7. . 8006935 10.1099/00222615-40-6-424

