# Supplementary figures and images for: Isolation of Multidrug-Resistant Escherichia coli O157 from Goats in the Somali Region of Ethiopia: A Cross-Sectional, Abattoir-Based Study
Source: PLoS One. 2015 Nov 11;10(11):e0142905. doi: 10.1371/journal.pone.0142905 (PMC4641637; doi:10.1371/journal.pone.0142905)

# Administrative Regions of Ethiopia

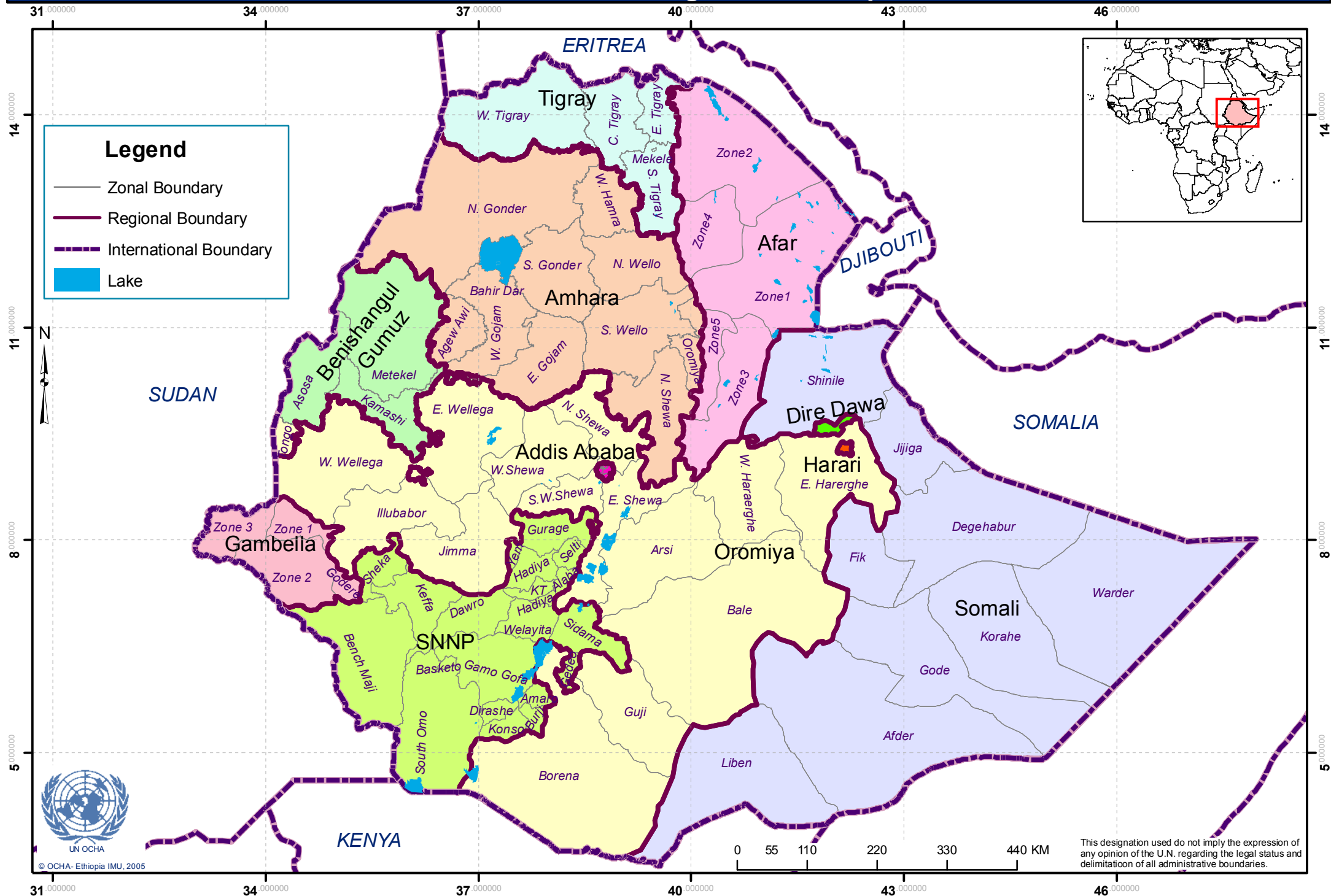

Supplement: S1 Fig — (PDF) [file pone.0142905.s001.pdf]
